# From Solid-Solution Strengthening to Grain Boundary Segregation: A Study on the Mechanism of Magnetic Property Evolution in Ni-Doped Fe-5.5Si Soft Magnetic Composites

**DOI:** 10.3390/mi17070852

**Published:** 2026-07-17

**Authors:** Xianjin Lan, Jiangyifan Wang, Ligang Liu, Yuanlin Xu, Chaojie Yang, Min Zhang

**Affiliations:** 1 School of Physics and Astronomy, China West Normal University, Nanchong 637002, China; 2 Sichuan Dongge Technology Co., Ltd., Nanchong 637676, China

**Keywords:** soft magnetic composites (SMCs), Fe-Si alloy, Ni doping, Cr doping, interface engineering, high-frequency properties

## Abstract

This study systematically investigates the effects of varying Ni doping levels (1.0–7.0 wt.%) on the microstructure, static magnetic properties, and high-frequency dynamic magnetic performance of Fe-5.5 wt.% Si soft magnetic composites (SMCs). Toroidal core samples were fabricated using powder metallurgy combined with silicone resin coating and high-temperature annealing. The influence of Ni doping on phase composition, morphology, saturation magnetization, coercivity, effective permeability, quality factor, total core loss and its components, and DC bias characteristics was comprehensively evaluated by XRD, SEM, EDS, hysteresis loop testing, and DC bias measurements. The results indicate that an appropriate Ni content (3.0–5.0 wt.%) promotes the formation of α-Fe(Si,Ni) solid solution and (Fe,Ni)_3_Si ordered phases, optimizes grain size and structural ordering, enhances saturation magnetization, and reduces coercivity. In contrast, excessive Ni doping (7.0 wt.%) leads to Ni segregation at grain boundaries, forming strong pinning centers that significantly increase coercivity and hysteresis loss. Within the wide frequency range of 1–100 kHz, Ni doping improves the permeability retention under DC bias but reduces the initial effective permeability. Notably, the sample with 5.0 wt.% Ni exhibits the highest quality factor (Q value) across the entire frequency range, demonstrating the best overall performance. This study provides experimental evidence and theoretical guidance for developing high-saturation-resistance, low-loss soft magnetic composites for medium-to-high-frequency applications.

## 1. Introduction

The rapid evolution of smart grids and high-frequency power converters has pushed the performance envelope of magnetic cores far beyond traditional 50/60 Hz designs. Within this landscape, the Fe-5.5Si alloy has secured a practical niche as a medium-to-high-frequency soft magnetic materials, largely because it strikes a commercially viable balance between saturation induction, resistivity, and cold workability [1]. Unlike higher-Si grades (e.g., Fe-6.5Si) that suffer from severe brittleness and processing difficulties, Fe-5.5Si maintains adequate workability for powder processing while providing sufficient resistivity for medium-to-high-frequency applications. Its commercial availability as gas-atomized powders also enables systematic alloying studies on an industrially relevant platform. Yet as operating frequencies climb toward 100 kHz and power densities intensify, even this workhorse composition begins to show its limits. Core losses surge, permeability collapses under DC bias, and the quality factor degrades faster than circuit designers would tolerate [2].

Micro-alloying is an effective approach for optimizing the performance of SMCs. Ni, as a ferromagnetic element, has been extensively studied in various soft magnetic systems. In Fe-Si-Nb-Cu-B amorphous/nanocrystalline alloys, Ni addition primarily serves to modulate nanocrystallization behavior and influence grain size and soft magnetic properties [3,4]. In Fe-Ni-based permalloy alloys, high Ni content yields exceptionally high initial permeability, mainly due to the significant reduction of magnetocrystalline anisotropy and magnetostriction by Ni [5]. Furthermore, in Fe-Si-based SMCs, the introduction of Ni coating or alloying has been confirmed to effectively increase electrical resistivity, reduce eddy current loss, and improve the quality factor (Q value) [6]. Studies have shown that the quality factor of Fe-Si-Ni magnetic powder cores prepared by powder metallurgy increases with higher Ni content, while core losses decrease accordingly. Xu et al. fabricated SMCs using Fe-Si-Ni powders via ball milling, phosphoric acid surface passivation, and optimized compaction processes, achieving a core loss as low as 285 mW/cm^3^ at 50 kHz/50 mT and a permeability retention exceeding 70% under a DC bias field of 50 Oe [7]. Zhao et al. coated FeSi SMCs with FeNi nanoparticles, successfully improving permeability and loss performance. With the addition of 15 wt.% FeNi nanoparticles, the effective permeability increased by 43.8% and core loss decreased by 22.1% [8]. Bahrami et al. prepared nanocrystalline Fe-Ni-Si soft magnetic powders by mechanical alloying, revealing that increasing Si content refines grain size and enhances lattice strain while reducing saturation magnetization; coercivity first increases and then decreases with ball milling time, closely related to the evolution of grain size [9]. Chen et al. designed a quaternary Fe-Si-Co-Ni soft magnetic alloy and demonstrated that an appropriate amount of Ni doping promotes the formation of the D0_3_ ordered phase, reduces magnetocrystalline anisotropy, and optimizes the magnetic domain structure. The resulting SMC exhibited a high effective permeability (*μ_e_*) of 160.6, a low core loss of 288.4 mW/cm^3^ at 100 kHz/50 mT, and a saturation magnetization of 177.3 emu/g, outperforming conventional Fe-based SMCs [10]. Wan et al. employed first-principles calculations to investigate the interaction between Ni and Si atoms within the α-Fe lattice, confirming a strong attractive interaction between Ni and Si, which facilitates the formation of stable Ni-Si atomic clusters and thereby modulates the lattice ordering and atomic arrangement of Fe-Si alloys [11]. Krishnan et al. found that substitutional doping of Ni in FeSi alloys introduces impurity bands near the Fermi level, significantly altering the band structure and resistivity characteristics [12]. Collectively, these studies indicate that Ni can exert multiple beneficial effects in Fe-Si-based systems: lattice modulation through substitutional solid solution, promotion of ordered D0_3_-type phases, enhancement of electrical resistivity, and direct contribution of ferromagnetic moments when properly incorporated. However, the efficacy of these mechanisms is strongly dependent on Ni content, as excess Ni may segregate and introduce detrimental pinning effects.

Nevertheless, existing research has mainly focused on amorphous/nanocrystalline systems, high-Ni-content Fe-Ni alloys, or compositionally complex multi-component SMCs. The mechanism by which Ni incorporation regulates intrinsic magnetic properties through ordered structure modulation in Fe-Si alloys remains insufficiently explored. Although several studies have reported the performance of Fe-Si-Ni composites at specific frequencies, no systematic experimental investigations have yet been conducted on how trace Ni doping quantitatively affects the complex permeability spectra, core loss, quality factor (Q value) frequency characteristics, and permeability retention under DC bias for Fe-5.5Si alloys across a wide frequency range (1–100 kHz).

Therefore, this study aims to systematically investigate the regulatory effect and underlying mechanism of trace Ni addition on the magnetic properties of crystalline Fe-5.5Si alloys. By comprehensively measuring the complex permeability, total core loss and loss components, quality factor spectrum, and DC bias characteristics within the frequency range of 1–100 kHz, a complete high-frequency dynamic performance will be established. Ultimately, the correlations between Ni content, lattice structure and ordering, high-frequency loss, and DC bias performance will be elucidated, providing a solid experimental and theoretical foundation for the development of high-performance, low-cost medium-to-high-frequency SMCs.

The downsizing of high-frequency power converters places stringent demands on passive magnetic components, as magnetic cores often constitute the largest volume in a converter and dominate overall power loss. In compact planar transformers, embedded inductors, and EMI filters for integrated power modules, core loss has become a primary bottleneck for increasing power density [13]. Modern GaN HEMTs now switch at frequencies exceeding 1 MHz, yet commercially available magnetic cores suffer from severe degradation of permeability and rapid escalation of core loss above 100 kHz, limiting the efficiency advantage of wide-bandgap devices [14]. Emerging solutions including embedded planar power inductors and on-chip integrated magnetics rely fundamentally on soft magnetic composites (SMCs) with tailored high-frequency responses and compatible powder-metallurgy processing routes [15]. Thus, engineering the composition and microstructure of crystalline Fe-Si alloys via trace Ni addition could provide a viable and scalable route to miniaturized, high-efficiency magnetic components for next-generation micro-power systems [16].

## 2. Experiments

### 2.1. Material Preparation

According to the nominal compositions of Fe-5.5Si-xNi (x = 1.0, 3.0, 5.0, or 7.0 wt.%), spherical Fe-5.5Si powders prepared by gas atomization (D50 = 15.16 μm) and high-purity nickel powders (purity: 99.99%, average particle size < 0.5 μm) were weighed and then mixed and ground for 10 min. Subsequently, the commercially available SH-9601 methylphenyl polysiloxane silicone resin (solid content: 50 ± 1%, purchased from Hubei Longsheng Sihai Material Co., Ltd., Zaoyang, China) was completely dissolved in acetone at a mass ratio of 1:10, followed by ultrasonication for 10 min to obtain a homogeneous resin solution. The as-obtained Fe-5.5Si-xNi mixed powders were then mixed with the resin solution to achieve a resin content of 1 wt.% relative to the total powder mass. The mixture was continuously stirred until complete evaporation of acetone, after which the powder was dried in an oven at 70 °C for 3 h to remove residual solvent. Zinc stearate (0.5 wt.%) was added to the dried powder as a lubricant and mixed uniformly. The mixture was uniaxially pressed at 900 MPa at room temperature into toroidal green compacts (outer diameter 15 mm, inner diameter 10 mm, height 10 mm). The green compacts were annealed at 880 °C for 5 h under an argon atmosphere, finally yielding Fe-5.5Si-x wt.% Ni (x = 1.0, 3.0, 5.0, 7.0) soft magnetic composites.

### 2.2. Characterization and Testing Methods

The phase composition of the samples was analyzed by X-ray diffraction (XRD, TD-3500, Dandong Tongda Science & Technology Co., Ltd., Dandong, China). Quantitative phase analysis was performed using the Rietveld full-spectrum fitting method. This approach employs GSAS software (GSAS-II) to fit the complete diffraction pattern via least squares, with refinement quality evaluated based on criteria such as R_wp_ (weighted residual factor) and χ^2^ (fit goodness). The microstructures of the powders and composites were observed using scanning electron microscopy (SEM, Hitachi Regulus 8230, Tokyo, Japan), and the major and minor elemental compositions of the alloy powders were determined by energy-dispersive X-ray spectroscopy (EDS). The resistivity measurement was performed using the four-probe method (ST2258C, Suzhou Jingge Electronic Co., Ltd., Suzhou, China). Static hysteresis loops of the samples were measured using a NIM-62000 hysteresis loop tester to obtain parameters such as saturation magnetization and coercivity. The hysteresis loss of the SMCs was measured using a soft magnetic AC current analyzer (MAST-3010SA, Hunan Linkjoin Technology Co., Ltd., Luodi, China). The DC bias characteristics and effective permeability *μ_e_* of the SMCs were analyzed using an LCR meter combined with a DC bias source (TH1778A, Changzhou Tonghui Electronics Co., Ltd., Changzhou, China). The effective permeability was calculated according to the formula $\mu_{e}=\frac{L_{S}l_{e}}{\mu_{0}N^{2}A_{e}}$, where *μ*_0_ is the vacuum permeability, *L_s_* is the inductance, *A_e_* is the effective cross-sectional area, *l_e_* is the effective magnetic path length, and *N* is the number of turns of the wire wound around the sample. For the measurements, toroidal samples were wound with 17 turns of copper wire with a diameter of 0.5 mm.

## 3. Results and Discussion

**Figure 1:**
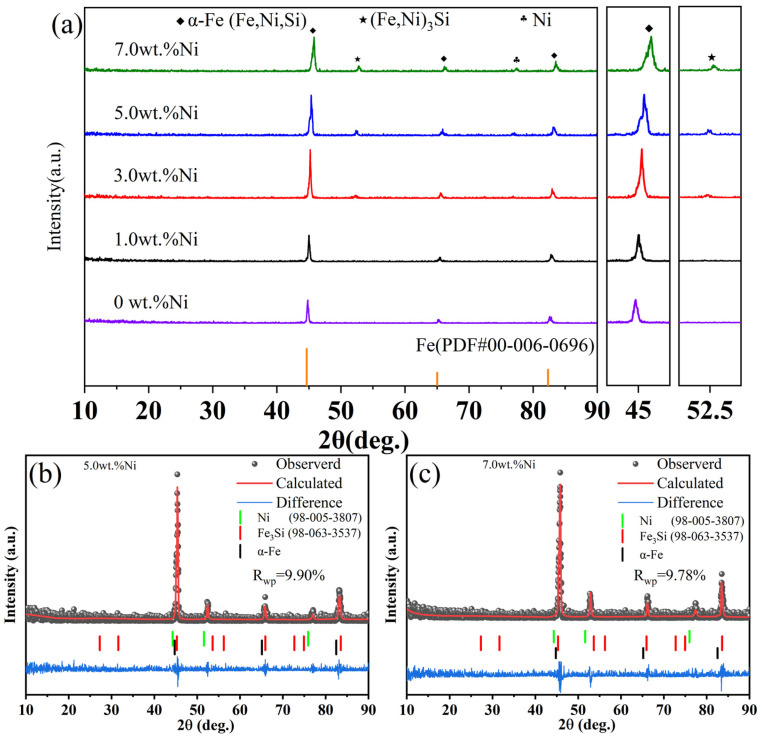
(**a**) X-ray diffraction patterns of Fe-Si raw material powder and magnetic core blocks; the inset shows the actual appearance of the FeSiCr sample; Rietveld refinement of XRD patterns and crystal structure diagrams for (**b**) 5.0 wt.%Ni and (**c**) 7.0 wt.%Ni.

Figure 1a shows the XRD patterns of Fe-5.5Si SMCs with varying Ni doping contents (x = 1.0–7.0 wt.%). The main diffraction peaks of all samples correspond to the α-Fe(Si) phase [17]—i.e., a body-centered cubic (BCC) substitutional solid solution in which Si atoms occupy Fe lattice sites—indicating that most Ni atoms have been successfully incorporated into the α-Fe(Si) lattice as a solid solution, forming an α-Fe(Si,Ni) solid solution. With increasing Ni doping content, the intensity of the principal diffraction peak near 45° increases significantly. It is important to note that this peak corresponds to the (110) plane of the body-centered cubic (BCC) α-Fe(Si) matrix, rather than the (200) plane. Simultaneously, this principal peak exhibits a systematic slight shift toward higher angles, indicating lattice contraction due to the partial substitution of Fe by the smaller Ni atoms.

It is worth noting that the diffraction peak near 45°does not exhibit a perfectly symmetric single-peak profile in the 5.0 wt.% and 7.0 wt.% Ni sample, where a splitting is observed on the lower-angle side. Based on Rietveld refinement, this asymmetry is attributed to the overlapping of three closely spaced reflections: (i) the (110) peak of the BCC α-Fe(Si,Ni) solid solution (~44.6–44.8°), (ii) the (111) peak of the precipitated elemental Ni phase (~44.5°), and (iii) the (220) peak of the ordered (Fe,Ni)_3_Si phase (~45.1°). At low Ni doping (1.0 wt.% and 3.0 wt.%), most Ni atoms are incorporated into the α-Fe lattice; thus, the peak mainly shows asymmetric broadening. As the Ni content increases to 5.0 wt.%, the excess Ni exceeds the solid solubility limit and precipitates as the elemental Ni phase, leading to a more resolved double-peak feature. This assignment is strongly supported by the Rietveld quantitative phase analysis presented in Figure 1b,c.

This shift arises because the atomic radius of Ni (≈0.124 nm) is smaller than that of Fe (≈0.127 nm); when Ni partially substitutes Fe in the lattice, lattice contraction occurs, leading to a decrease in the interplanar spacing d. Such a peak shift to higher angles due to differences in solute atomic radii has also been observed in high-entropy alloy systems such as Fe-Co-Ni-Si-Al [18]. For samples with Ni doping contents ranging from 3.0 to 7.0 wt.%, a diffraction peak appears near 2θ ≈ 52°, which corresponds to the Fe_3_Si phase (D0_3_-type cubic structure, space group Fm-3m). The intensity of this peak gradually increases with Ni content, accompanied by a pronounced shift to higher angles (2θ from 52.3° to 52.7°), indicating that additional Ni atoms further participate in the formation of an ordered (Fe,Ni)_3_Si solid solution [19]. These peak shifts directly demonstrate that Ni is dispersed in the Fe-Si matrix in the form of α-Fe(Si,Ni) and (Fe,Ni)_3_Si solid solutions.

Figure 1b,c present the Rietveld-refined XRD patterns for the 5.0 wt.% and 7.0 wt.% Ni-doped samples, respectively. Quantitative analysis confirmed the coexistence of three phases in these samples: α-Fe(Si,Ni) solid solution, ordered (Fe,Ni)_3_Si phase (gupeiite), and Ni phase. In the 5.0 wt.% Ni sample, the phase fractions are 88.3 wt.% α-Fe, 10.2 wt.% Ni, and 1.5 wt.% (Fe,Ni)_3_Si; increasing Ni to 7.0 wt.% shifts these to 81.7 wt.%, 15.9 wt.%, and 2.3 wt.%, respectively. The lattice constants obtained from refinement indicate that the α-Fe phase has a lattice constant of approximately 2.864–2.865 Å, the Ni phase approximately 3.531–3.532 Å, and the (Fe,Ni)_3_Si phase approximately 5.640 Å in both samples. Lattice strain analyses show that for the 5.0 wt.% Ni sample, the strains in α-Fe, Fe_3_Si, and Ni are 0.29%, 0.30%, and 0.28%, respectively. For the 7.0 wt.% Ni sample, the corresponding strains are 0.31%, 0.30%, and 0.25%. The slightly larger strain in α-Fe at higher Ni doping suggests that more Ni in solid solution causes greater lattice distortion. This strain increase likely arises from two coupled effects: atomic size mismatch as Ni substitutes for Fe, and the emergence of excess Ni precipitates once solubility is exceeded, which introduce additional coherent/semi-coherent strain fields at the matrix-precipitate interfaces.

**Figure 2:**
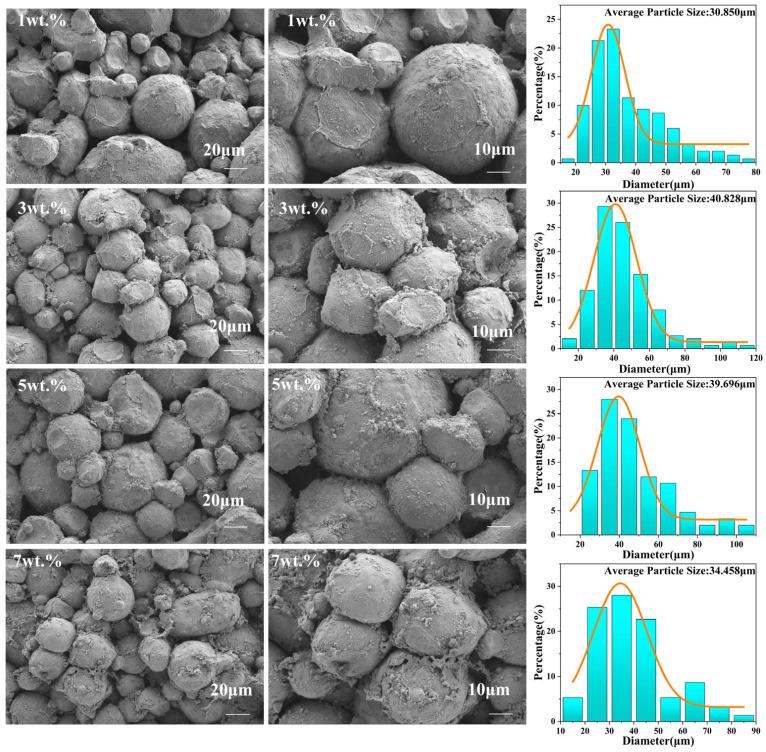
SEM images of Fe-5.5Si SMCs doped with Ni.

Figure 2 shows the SEM microstructures of Fe-5.5Si soft magnetic composites with varying Ni doping contents (x = 1.0–7.0 wt.%) after pressing and sintering. All samples exhibit a spherical-like particle morphology with rough and dense surfaces. A uniform adherent layer is observed covering the particle surfaces, which is consistent with previous reports [20], indicating that the introduction of Ni does not significantly alter the basic morphological characteristics of the powder particles. However, with increasing Ni doping content, the particle size distribution shows a tendency toward refinement. Notably, in the sample with 5 wt.% Ni, the proportion of small-sized particles increases significantly (see Table 1). This particle refinement phenomenon is consistent with the trend in sample density, suggesting that the addition of Ni may inhibit the agglomeration or growth of particles during pressing or sintering [21].

**Figure 3:**
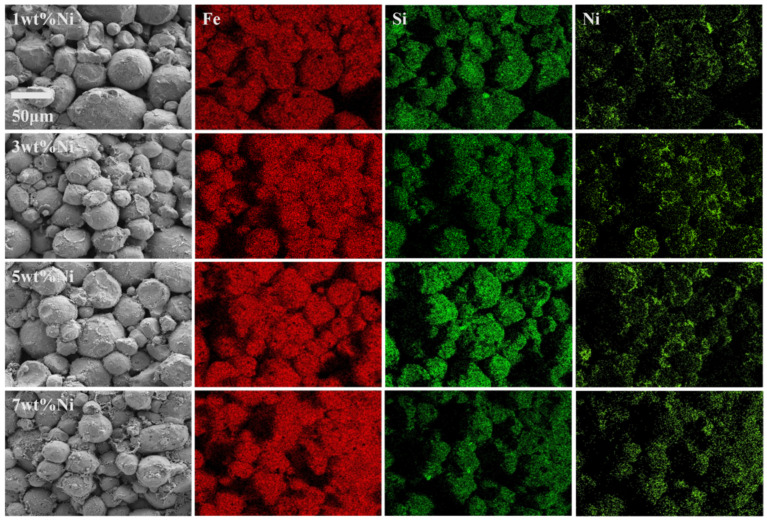
EDS elemental distribution maps of Fe-5.5Si SMCs with different Ni doping contents.

Figure 3 shows the microstructure and EDS elemental mapping of Fe-5.5Si SMCs, with the corresponding elemental compositions listed in Table 2. It should be noted that the EDS mapping on rough particle surfaces is qualitatively indicative but suffers from topographic effects and detector shadowing, which may artificially enhance local signal contrasts. Therefore, the mapping is used here only to illustrate the general elemental distribution trend. The Fe signal appears relatively uniform across the scanned area, while Ni and Si show certain localized enrichment in specific regions (e.g., particle surfaces or inter-particle boundaries). The compositional fluctuations observed in EDS are qualitatively consistent with the phase fractions obtained from Rietveld analysis. The localized enrichment of silicon is likely attributable to the non-uniformity of the silicone resin coating. Notably, in the sample with x = 5 wt.% Ni, the Si content is relatively higher, and the silicone resin coating layer appears more continuous and complete. Although the measured contents of Fe and Si fluctuate to some extent after doping, the overall variation is relatively small, and no proportional decrease with increasing Ni content is observed. This suggests that Ni atoms do not simply dilute the matrix; instead, they likely substitute Fe atoms into the lattice to form a substitutional (Fe,Ni)_3_Si solid solution, thereby redistributing the elemental proportions. This compositional change pattern is consistent with previous findings that Ni tends to occupy specific lattice sites and form solid solutions in Fe_3_Si alloys [22]. In contrast, the distribution of Ni exhibits non-uniform characteristics. As the nominal Ni doping content (x) increases from 1.0 wt.% to 7.0 wt.%, the Ni content measured by EDS shows a monotonic increasing trend (see Table 2), indicating that Ni has been successfully incorporated into the material, with the actual content variation following the same trend as the nominal addition [23]. Meanwhile, with increasing Ni content, local enrichment regions of Ni appear on the particle surfaces and interparticle regions. Combined with the XRD results, it can be inferred that in samples with low Ni doping levels, the secondary phase does not precipitate extensively as elemental Ni, but rather segregates as (Fe,Ni)_3_Si solid solution on particle surfaces or at grain boundaries. In contrast, samples with high Ni doping levels exhibit the presence of elemental Ni phase.

**Figure 4:**
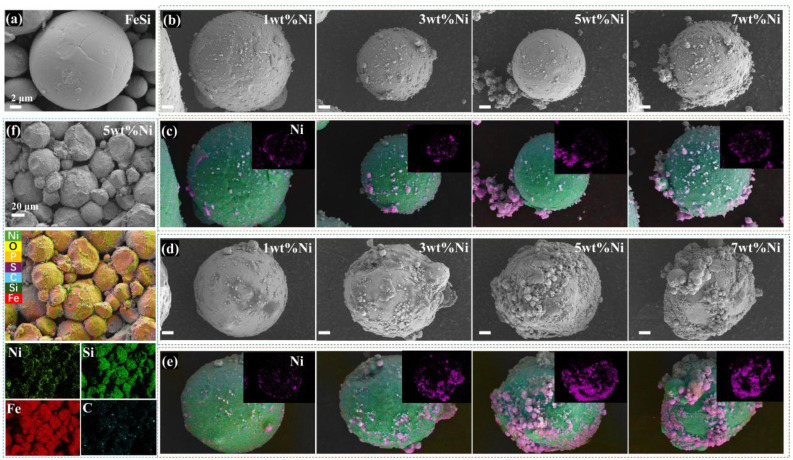
SEM images of magnetic core powder particles: (**a**) particle morphology of raw FeSi powder; (**b**,**c**) particle morphology and elemental distribution of Ni-doped FeSi powder, with an inset showing Ni distribution; (**d**,**e**) particle morphology and elemental distribution of FeSi powder after addition of silicone resin, with an inset showing Ni distribution; (**f**) sintered FeSi-5Ni soft magnetic core.

The insulating layer on the surface of FeSi particles can effectively isolate metallic particles and suppress high-frequency eddy current losses. To confirm whether the FeSi particles successfully achieved coating after Ni doping and silicone resin treatment, the morphology and elemental distribution before and after interface improvement of the magnetic core powder particles were further compared, as shown in Figure 4. Figure 4a shows the FeSi raw powder particles, which exhibit a spherical shape with relatively uniform size, a smooth and dense surface, and fine pits. After the addition of Ni powder, Ni adheres to the particle surfaces; with increasing Ni content, the surface roughness significantly increases and surface deposits form, as illustrated in Figure 4b,c. Following the addition of the silicone resin, as the Ni doping content increases, the particle surface gradually transitions from partial coverage and pit filling to being wrapped by a continuous and dense organic layer, with the original spherical contour becoming blurred (Figure 4c,e). This confirms that the organic coating layer effectively fills the surface micropores, enhancing interfacial bonding and providing insulation. After pressing and high-temperature sintering, distinct sintering necks form between the particles (see Figure 4f), and the spherical particle morphology disappears as the particles fuse into a dense bulk structure. In the 5 wt.% Ni sample, both carbon (C) and nickel (Ni) exhibit certain segregation between particles. The presence of C, originating from the silicone resin, confirms the formation of an insulating resin coating layer on the particle surfaces, while Ni-rich regions indicate the precipitation of elemental Ni phase. The insulating layer on the particle surface is critical for suppressing high-frequency eddy current losses. While the SEM images in Figure 4 show that the silicone resin covers the particle surfaces and fills surface micropores, morphology alone is insufficient to confirm electrical insulation. To verify this, we measured the DC volume resistivity of the sintered toroidal cores. The Fe-5.5Si-5Ni sample exhibits a resistivity of 689.7 μΩ·cm, which is significantly higher than that of bare Fe-Si alloys (74 μΩ·cm) [24], confirming that the silicone resin coating effectively creates an insulating barrier between adjacent metallic particles.

**Figure 5:**
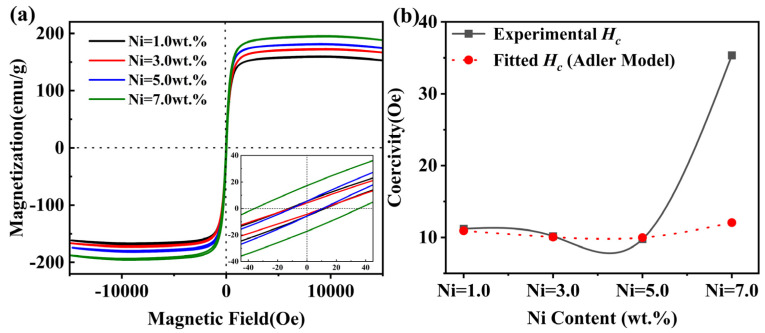
(**a**) The hysteresis loops of Fe-5.5Si SMCs doped with different Ni concentrations, with an inset providing an enlarged view near the zero-field region; (**b**) Fitting results of the Adler model for coercive force.

Figure 5a shows the hysteresis loop of Fe-5.5Si SMCs doped with different Ni concentrations. All samples display typical characteristics of soft magnetic materials, namely narrow and steep hysteresis loops with extremely low remanence and coercivity, indicating good soft magnetic performance. The inset of Figure 5a presents a magnified view of the low-field region of the hysteresis loops for Fe-5.5Si SMCs with varying Ni doping contents, and the corresponding magnetic parameters are summarized in Table 3.

The saturation magnetization *M_s_* gradually increases with increasing Ni content. The increase in *M_s_* can be attributed to two main factors. First, Ni itself is a ferromagnetic element; its solid solution into the α-Fe(Si) lattice directly contributes atomic magnetic moments [25]. Second, the introduction of trace amounts of Ni may optimize the alignment of magnetic moments in the matrix by influencing short-range ordering or inhibiting the formation of brittle ordered phases (such as B2 or D0_3_) [26,27]. Literature indicates that Fe_3_Si itself is a ferromagnetic phase with relatively high saturation magnetization. With appropriate Ni content (3.125 at.%), (Fe,Ni)_3_Si formed by partial substitution of Fe by Ni may exhibit an ordered structure favorable for parallel alignment of magnetic moments, thereby achieving higher saturation magnetization than pure Fe_3_Si or α-Fe(Si) solid solution [28].

Table 3 lists the magnetic parameters of Fe-5.5Si SMCs at different Ni doping concentrations. Combined with the XRD analysis in Figure 1, this data provides a comprehensive understanding of the microscopic mechanisms underlying how magnetic properties vary with Ni content. As the Ni content increased from 1.0 wt.% to 5.0 wt., *M_s_* rose steadily from 161.22 emu/g to 182.26 emu/g, while the coercivity *H_c_* decreased gradually from 11.21 Oe to 9.76 Oe. Within this range, XRD results demonstrated that Ni atoms were successfully solubilized into the α-Fe(Si) lattice, forming an α-Fe(Si,Ni) solid solution. This solid-solution strengthening effect optimized the magnetic domain structure and reduced magnetic crystal anisotropy, thereby simultaneously enhancing *M_s_* and lowering *H_c_*.

However, when the Ni content is further increased to 7.0 wt.%, the magnetic properties exhibit a significant change. Rietveld refinement analysis reveals the underlying cause of this anomaly. Beyond the solid solubility limit of Ni doping, excess Ni precipitates as the elemental Ni phase and the ordered (Fe,Ni)Si phase. The introduction of these non-magnetic or weakly magnetic secondary phases, particularly the ordered (Fe,Ni)Si_3_ phase, significantly enhances magnetic crystal anisotropy and the pinning effect, resulting in substantial increases in both *H_c_* and *M_r_*. Therefore, moderate Ni doping (≤5.0 wt.%) effectively improves the soft magnetic properties of Fe–5.5Si powder, whereas excessive doping (≥7.0 wt.%) severely deteriorates these properties due to secondary phase precipitation.

The remanent magnetization *M_r_* exhibits a non-monotonic increasing trend, rising from 5.59 emu/g to 17.15 emu/g, indicating that Ni addition enhances the material’s ability to retain remanence. This phenomenon may be related to the modification of the exchange coupling effect in the ferromagnetic matrix by Ni atoms [29].

The coercivity *H_c_* exhibits a non-monotonic trend with increasing Ni content, first decreasing and then increasing. When the Ni content reaches 7.0 wt.%, *H_c_* sharply increases to 35.36 Oe, which is more than three times that of the sample with 1.0 wt.% Ni. This sharp rise is likely related to Ni agglomeration. When the Ni doping level exceeds its solid solubility limit in the Fe-Si matrix [30], the excess Ni cannot fully enter the α-Fe(Si) lattice or form the ordered (Fe,Ni)_3_Si phase, leading to segregation of surplus Ni atoms. The Ni agglomerates act as strong pinning sites, thereby increasing the coercivity [31]. According to the coercivity model for soft magnetic alloys proposed by Adler et al. [32], *H_c_* can be described by the grain size *d_K_*, saturation magnetization *M_s_*, and domain wall energy γ_w_, as follows:

$$H_{c}=\frac{15}{16}\pi \frac{\gamma_{w}}{\mu_{0}M_{s}}\cdot \frac{1}{d_{K}}$$

Based on this formula, combined with the microstructures shown in Figure 2 and the grain sizes listed in Table 1, Figure 5b is plotted. For the samples with 1.0–5.0 wt.% Ni, the fitted coercivity values are in good agreement with the experimental results, validating the applicability of the Adler model within this low-doping range. For the sample with 7.0 wt.% Ni, the experimental coercivity (35.36 Oe) is much higher than the fitted value (12.06 Oe). Combined with the EDS elemental mapping results, this confirms that the excess Ni segregates at grain boundaries, forming strong domain wall pinning centers, which become the dominant factor responsible for the sharp increase in coercivity.

**Figure 6:**
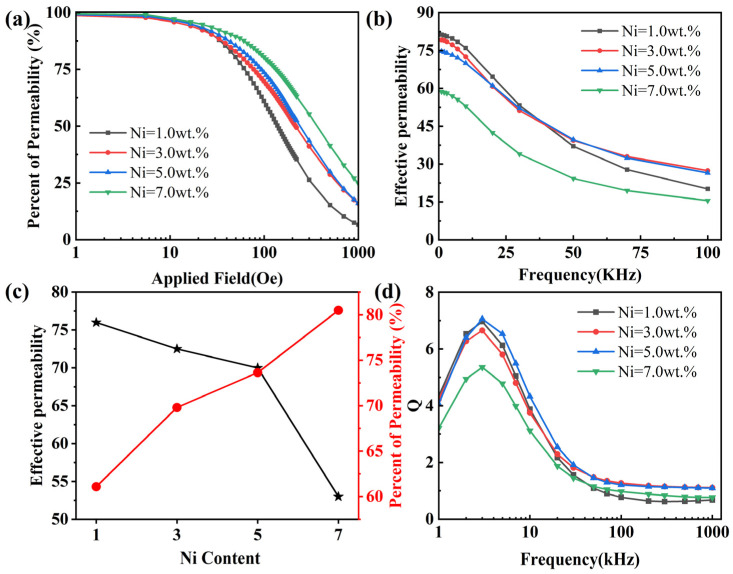
(**a**) DC bias curves of Fe-5.5Si SMCs doped with different Ni contents; (**b**) Variation curve of effective permeability versus frequency; (**c**) Dependence plots of DC bias and effective permeability on Ni content; (**d**) Variation curve of quality factor Q versus frequency.

Figure 6a illustrates the variation in relative permeability in FeSi soft magnetic composites (SMCs) under different Ni doping levels with respect to the applied DC bias magnetic field. As the external magnetic field increases, the relative permeability of all samples shows a declining trend. In the low-field region (<10 Oe), the permeability curves of all samples nearly overlap, indicating similar initial magnetization behaviors. With further increase in the DC bias field, the permeability of all sample compositions exhibits a monotonic decrease, attributed to gradual saturation pinning of magnetic domain walls and competition between reversible and irreversible magnetization processes. In the high-field region (>100 Oe), Ni incorporation significantly enhances the material’s anti-saturation capability; specifically at 1000 Oe, samples with 7.0 wt.% Ni retain approximately 25% of their initial permeability, whereas those with 1.0 wt.% Ni show only about 8% retention. This result aligns with observations from FeSiBPNb amorphous magnetic powder cores treated with FeSi powder, demonstrating that high-saturation components effectively improve magnetic stability under strong DC fields [33].

Figure 6b shows the variation in effective permeability *μ_e_* with frequency (f) for samples with different Ni contents. The *μ_e_* values of all samples gradually decrease with increasing frequency, reflecting a typical magnetic dispersion behavior in soft magnetic composites, primarily due to the combined effects of hysteresis effects in domain wall motion at high frequencies, eddy current shielding, and ferromagnetic resonance absorption [34]. When Ni content is maintained at low levels (1.0–5.0 wt.%), the samples exhibit high initial effective permeability with relatively gentle frequency-dependent decay, demonstrating excellent high-frequency stability. However, when Ni content increases further to 7.0 wt.%, the initial effective permeability drops significantly to approximately 59, with the most pronounced attenuation observed across the entire measurement range—reaching only about 15 at 100 kHz. This phenomenon arises from two synergistic mechanisms: first, excess Ni increases the alloy’s magnetic crystal anisotropy constant, raising the potential barrier for domain rotation and reducing initial permeability; second, elevated Ni content may alter exchange coupling interactions between ferromagnetic particles, thereby enhancing eddy current losses and exacerbating permeability decay at high frequencies.

Figure 6d presents the variation in quality factor Q with frequency. Defined as the ratio of the real part (*μ*′) to the imaginary part (*μ*″) of complex permeability (Q = *μ*′/*μ*″), Q quantifies the relationship between magnetic core energy storage and energy loss per unit cycle, serving as a key comprehensive indicator for evaluating high-frequency performance of soft magnetic materials [35]. All samples exhibited a Q value that first increased and then decreased, rapidly rising to peak values at low frequencies before gradually declining with increasing frequency, stabilizing above 100 kHz. Samples with Ni content of 5.0 wt.% maintained the highest Q values across the entire frequency range, demonstrating superior overall high-frequency performance. However, when Ni content increased to 7.0 wt.%, the Q values significantly declined throughout the spectrum, indicating that excess Ni exacerbates magnetic losses or eddy current losses, thereby elevating high-frequency energy dissipation. This finding aligns with previous studies on FeSi/FeNi composite magnetic cores reporting substantial increases in high-frequency losses due to excessive component concentrations [36]. These results demonstrate that moderate Ni addition (3.0–5.0 wt.%) enhances both initial permeability and Q values while improving DC bias resistance, whereas excessive Ni (7.0 wt.%) maximizes DC bias performance at the expense of high-frequency effective permeability and Q values.

These results demonstrate that moderate Ni addition (3.0–5.0 wt.%) enhances both initial permeability and Q values while improving DC bias resistance. Specifically, although the 3.0 wt.% Ni sample exhibits slightly better static properties, the 5.0 wt.% Ni sample outperforms it in terms of Q-factor above 10 kHz (Figure 6d) and DC-bias retention (Figure 6a), making 5.0 wt.% the optimal composition for high-frequency applications. Conversely, excessive Ni (7.0 wt.%) maximizes DC bias performance at the expense of high-frequency effective permeability and Q values.

**Figure 7:**
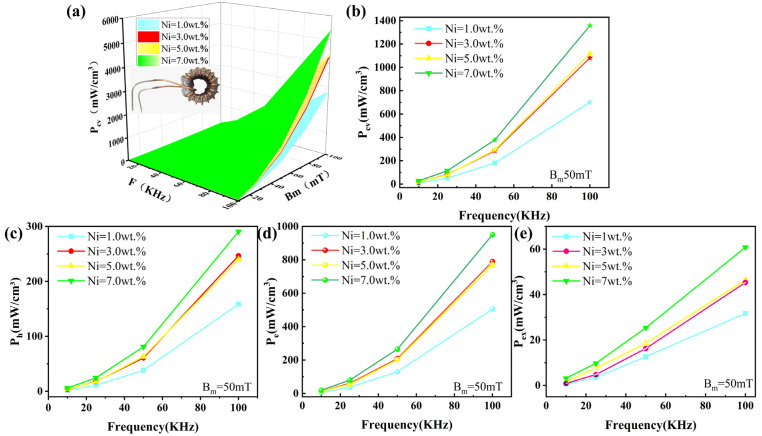
Loss characteristics of Fe-5.5Si soft magnetic composites with different Ni doping concentrations; (**a**) Three-dimensional surface plot showing total loss versus frequency and magnetic flux density; (**b**) Total loss *P_cv_*; (**c**) Hysteresis loss *P_h_*; (**d**) Eddy current loss *P_e_*; (**e**) Excess loss *P_ex_* as a function of magnetic field frequency at a magnetic flux density of 50 mT.

Figure 7a presents a three-dimensional total loss surface that intuitively reflects the significant increase in total core loss with increasing Ni content over a wide range of frequencies and magnetic flux densities. Particularly in the high-frequency (*f* > 100 kHz) and high magnetic flux density (*B_m_* > 50 mT) regions, the total loss of the sample with 7.0 wt.% Ni increases sharply, indicating extremely severe energy dissipation under high-frequency and high-induction operating conditions [37]. Figure 7b shows the variation in total loss *P_cv_* as a function of frequency f for the SMCs at *B_m_* = 50 mT. As the Ni doping content increases from 1.0 wt.% to 7.0 wt.%, the hysteresis loss under the same frequency and magnetic flux density conditions significantly increases [38]. This is primarily attributed to the Ni-induced increase in magnetocrystalline anisotropy or the introduction of microstructural defects, which increase the resistance to domain wall motion and magnetic moment rotation (i.e., increase coercivity) [37], thereby generating a larger hysteresis loop area during alternating magnetization and ultimately leading to a marked rise in hysteresis loss.

According to Bertotti’s loss separation theory, the total core loss *P_cv_* can be approximately decomposed into three components: hysteresis loss *P_h_*, eddy-current loss *P_e_*, and excess loss *P_ex_*, i.e., *P_cv_* ≈ *P_h_* + *P_e_* + *P_r_*. The loss separation can be performed using the following formula [39,40]:

$$P_{cv}\;=\;P_{h}\;+\;P_{e}\;+\;P_{ex}\;=\;K_{h}B_{m}^{a}f\;+\;K_{e}B_{m}^{2}f^{\;2}\;+\;K_{ex}B_{m}^{1.5}f^{\;1.5}$$

where the first, second, and third terms correspond to hysteresis loss, eddy current loss, and excess loss, respectively. $K_{h}$, $K_{e}$, and $K_{\mathrm{ex}}$ are the hysteresis, eddy-current, and excess loss coefficients, respectively. *B_m_* is the maximum magnetic flux density under an alternating magnetic field, and *f* is the frequency of the alternating magnetic field. Under low magnetic flux density and moderate frequency conditions, the residual loss is usually small and can be neglected.

As shown in Figure 7c, the sample with 1.0 wt.% Ni doping exhibits the lowest hysteresis loss *P_h_*, while the sample with 7.0 wt.% Ni shows the highest. Previous studies have demonstrated that *P_h_* is highly correlated with the coercivity of the material [41]. Trace Ni doping can optimize the internal magnetic domain structure of the alloy, reducing the pinning resistance to domain wall motion during magnetization, thereby decreasing coercivity and hysteresis loss. The 7.0 wt.% Ni represents excessive doping. EDS results reveal significant segregation of Ni at grain boundaries and particle surfaces, forming strong pinning centers. In this case, the Adler model is no longer applicable, and the coercivity *H_c_* sharply increases to 35.53 Oe, leading to the maximum hysteresis loss among all groups. In the eddy current loss *P_e_* curves shown in Figure 7d, *P_e_* increases with frequency *f* following a quadratic (*f*^2^) relationship, which is consistent with classical eddy current loss theory. With increasing Ni doping content, *P_e_* exhibits an increasing trend. This phenomenon may be attributed to the fact that Ni solid-solution strengthening alters the electrical conductivity or the surface insulation state of the powder particles, thereby deteriorating the overall eddy current loss.

## 4. Conclusions

This study prepared Fe-5.5Si annular magnetic core samples with varying Ni doping levels (1.0–7.0 wt.%) by combining powder metallurgy with silicone resin coating and high-temperature annealing processes. Using XRD, SEM, EDS, hysteresis loop testing, AC magnetometry analysis, and DC bias testing, the effects of Ni doping on the microstructure, static magnetic properties, and high-frequency dynamic magnetic performance of Fe-5.5Si SMCs were investigated. The main conclusions are as follows:

Microstructure and phase composition: Ni is successfully dissolved into the α-Fe(Si) lattice, forming an α-Fe(Si,Ni) solid solution, and promotes the formation of the ordered (Fe,Ni)_3_Si phase at higher Ni contents. Ni doping induces lattice contraction, as evidenced by the rightward shift in XRD peaks. Excess Ni (7.0 wt.%) segregates at grain boundaries and particle surfaces, resulting in the formation of elemental Ni.

Static magnetic properties: The saturation magnetization (*M_s_*) first increases and then decreases with increasing Ni content, reaching a maximum value of 283.67 emu/g at 3.0 wt.% Ni. The coercivity (*H_c_*) exhibits little variation at low Ni contents (≤5.0 wt.%) but sharply rises to 35.53 Oe at 7.0 wt.% Ni, which is attributed to the pinning centers formed by Ni segregation. The Adler model shows good agreement with experimental data at low Ni contents, confirming the dominant role of grain size in determining coercivity.

High-frequency dynamic performance: Ni doping significantly enhances the permeability retention under DC bias. The sample with 7.0 wt.% Ni achieves a retention rate as high as 25% at 1000 Oe, compared to only 8% for the 1.0 wt.% Ni sample. However, the initial effective permeability decreases with increasing Ni content, with the most pronounced decline observed in the 7.0 wt.% Ni sample. The sample with 5.0 wt.% Ni exhibits the highest quality factor (Q value) across the entire frequency range, demonstrating the best overall high-frequency performance.

Loss characteristics: The total core loss, hysteresis loss, and eddy current loss all increase with rising Ni content. The 7.0 wt.% Ni sample shows the highest losses under all frequency and magnetic flux density conditions, especially at high frequencies and high induction levels. The increased coercivity and microstructural deterioration caused by excess Ni are the primary reasons for the rise in hysteresis loss.

In summary, an appropriate amount of Ni doping (3.0–5.0 wt.%) significantly improves the DC bias resistance while maintaining relatively high initial permeability and low core loss. In contrast, excessive Ni doping (7.0 wt.%), despite its advantage in DC bias performance, severely compromises the high-frequency effective permeability and quality factor, leading to overall performance degradation.

## Figures and Tables

**Table 1:** Density average particle size (D), crystallite size (*d_k_*), and bulk resistivity of Fe-5.5Si SMCs with different Ni doping concentrations.

	*ρ* (g/cm^3^)	D (μm)	*d_k_* (nm)	*ρ_R_* (μΩ·cm)
Fe-5.5Si-1.0Ni	6.834	30.850 ± 0.804	33.4	857.6
Fe-5.5Si-3.0Ni	7.033	40.828 ± 0.827	44.1	710.6
Fe-5.5Si-5.0Ni	7.054	39.696 ± 1.483	32.6	689.7
Fe-5.5Si-7.0Ni	6.874	34.458 ± 1.682	29.0	469.4

**Table 2:** Element compositions of Fe-5.5Si SMCs doped with different Ni concentrations.

EDS	Fe (wt.%)	Si (wt.%)	Ni (wt.%)	C (wt.%)	O (wt.%)	S (wt.%)	P (wt.%)	Total
Fe-5.5Si-1.0Ni	67.58	5.24	4.47	13.90	8.76	0.02	0.03	100
Fe-5.5Si-3.0Ni	65.42	4.91	8.11	13.87	7.61	0.03	0.05	100
Fe-5.5Si-5.0Ni	67.67	5.03	8.74	12.41	6.09	0.03	0.03	100
Fe-5.5Si-7.0Ni	67.81	5.02	9.14	13.26	4.75	0.01	0.01	100

**Table 3:** Magnetic parameters of Fe-5.5Si powders doped with different Ni contents.

	*M_s_* (emu/g)	*H_c_* (Oe)	*M_r_* (emu/g)
Fe-5.5Si-1.0Ni	161.22	11.21	5.59
Fe-5.5Si-3.0Ni	172.83	10.18	5.16
Fe-5.5Si-5.0Ni	182.26	9.76	4.31
Fe-5.5Si-7.0Ni	196.57	35.36	17.15

## Data Availability

The original contributions presented in this study are included in the article. Further inquiries can be directed to the corresponding author.
